# The role of polymeric chains as a protective environment for improving the stability and efficiency of fluorogenic peptide substrates

**DOI:** 10.1038/s41598-022-12848-4

**Published:** 2022-05-25

**Authors:** Ana Arnaiz, Marta Guembe-García, Estefanía Delgado-Pinar, Artur J. M. Valente, Saturnino Ibeas, José M. García, Saúl Vallejos

**Affiliations:** 1 Departamento de Química, Facultad de Ciencias, Universidad de Burgos, Plaza de Misael Bañuelos s/n, 09001 Burgos, Spain; 2 CQC, Department of Chemistry, University of Coimbra, Rua Larga, 3004-535 Coimbra, Portugal

**Keywords:** Actuators, Sensors and biosensors

## Abstract

We have faced the preparation of fully water-soluble fluorescent peptide substrate with long-term environmental stability (in solution more than 35 weeks) and, accordingly, with stable results in the use of this probe in determining the activity of enzymes. We have achieved this goal by preparing a co-polymer of the commercial *N*-vinyl-2-pyrrolidone (99.5% mol) and a fluorescent substrate for trypsin activity determination having a vinylic group (0.5%). The activity of trypsin has been measured in water solutions of this polymer over time, contrasted against the activity of both the commercial substrate Z-L-Arg-7-amido-4-methylcoumarin hydrochloride and its monomeric derivative, prepared *ad-hoc*. Initially, the activity of the sensory polymer was 74.53 ± 1.72 nmol/min/mg of enzyme, while that of the commercial substrate was 20.44 ± 0.65 nmol/min/mg of enzyme, the former maintained stable along weeks and the latter with a deep decay to zero in three weeks. The ‘protection’ effect exerted by the polymer chain has been studied by solvation studies by UV–Vis spectroscopy, steady-state & time resolved fluorescence, thermogravimetry and isothermal titration calorimetry.

## Introduction

Pathogenic bacteria and viruses release a specific set of enzymes (named proteases) causing several dangerous conditions in different fields, e.g., biomedicine^1–6^, environmental microbiology^7–11^, and food safety^12,13^. For many years, the activity of different proteolytic enzymes (proteases) that are released during pathogenesis has been analysed by fluorogenic peptide substrates. These substrates are either small (e.g., 1 to 10 amino acids) or much larger peptides and contain motifs of fluorescent molecules (fluorophores) covalently anchored to the peptide. The fluorophores are combined with quenchers and give rise to systems based on non-radiative energy transfer processes, specifically, FRET processes (Förster/fluorescence resonance energy transfer). When a specific protease targets the fluorogenic peptide substrate, it cleaves the peptide and separates the fluorophore from the quencher, triggering fluorescence^14^. Thanks to the fluorescent easy understandable and highly specific mechanism, fluorogenic peptide substrates are used to perform fluorometric measurements in various valuable applications, such as studies of binding interactions, and determinations of enzymes’ catalytic activity^15–17^. The latter is the most common use, and many examples can be found in the literature, such as determination of catalytic activity of lysostaphin^18^, endopeptidases such as thermolysin, asparaginyl, or protealysin^19–21^, and endopeptidase matrix metalloproteases (MMPS) as MMP-2^22,23^.

Despite the advantages, the use of these fluorogenic peptide substrates is limited mainly for two reasons: 1) low stability at room temperature and refrigerated:^24^ they must be stored at − 20 °C, as indicated by the majority of manufacturers, and they exhibit a significant loss of activity after a few weeks, making this short shelf life a limitation for their use, especially when performing studies along time with the same batch; 2) lack of solubility in water:^25^ an organic solvent (such as DMSO) is usually required to prepare a concentrated substrate solution, which is then diluted in an aqueous-organic mixture. Therefore, when measurements are carried out with these systems, the medium in which the enzyme recognizes and cleaves the fluorogenic substrate is not the same as the biological medium (fully aqueous), which affects and changes the efficiency of the enzyme, and the veracity of the results.

Polymer science can be the effective solution to overcome some of fluorogenic peptide substrates drawbacks: 1) Regarding stability, VP was selected as the main monomer for the polymer synthesis, because the derived co-polymers are highly hydrophilic, non-fluorescent^26^, and have previously demonstrated protective effects toward sensitive chemical groups without the need of including spacers^27–35^; 2) regarding water solubility, we have previously described the preparation of polymers in 100% aqueous media in several publications^36–39^. These polymers contain a small proportion of receptor monomers originally water-insoluble (mol% < 1%). However, when co-polymerized with hydrophilic monomers, they give rise to hydrophilic materials that can be worked with in a 100% aqueous medium (membranes), or even to fully water-soluble polymers.

In this work we have designed and prepared a polymerizable substrate for trypsin (monomer substrate, MONO-SUBS) that can be part of a copolymer (mol% < 1%) in which the random coil safeguards the substrate generating a protective environment and allowing working in fully aqueous media (sensory material, POLY-SUBS). In addition, we have carried out a comparative stability study between them, including a reference/model substrate (SUBS, Z-L-Arg-7-amido-4-methylcoumarin)^40–42^. This substrate has a simple structure based on the fluorophore 7-amino-4-methylcoumarin (AMC)^43–45^ and an amino acid (L-arginine). Then, we have slightly modified its structure into a vinylic monomer (MONO-SUBS) by introducing in its structure a styrene motif. Next, we have prepared the water-soluble polymer (POLY-SUBS), which have active groups for the fluorogenic detection of trypsin, by radical initiated polymerization of *N*-vinyl-2-pyrrolidone (VP, 99.5 mol%) and a small proportion of MONO-SUBS (0.5 mol%).

**Figure 1:**
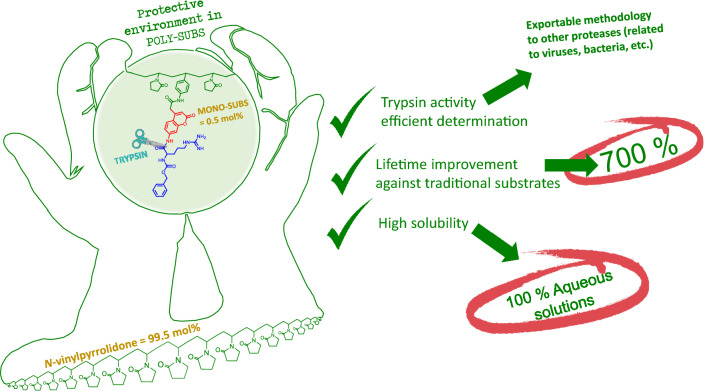
Graphical abstract of the study depicting the general idea, including the main advantages of the finding.

We believe that in the near future, this new methodology can be applied in the preparation of sensory materials (coatings, masks, surfaces, etc.) for the easy and visual detection of pathogens through their related proteases (Fig. 1)^46^.

## Materials and methods

### Materials

All materials and solvents were commercially available and used as received unless otherwise indicated. The following materials and solvents were used: *N*-vinyl-2-pyrrolidone (VP) (Acros Organic, 99%), 4-aminostyrene (TCI, 98%), hydrochloric acid (VWR-Prolabo, 37%), sodium hydroxyde (VWR-Prolabo, 99%), diethyl ether (VWR-Prolabo, 99%), dimethylformamide (Merck, 99%), ethanol (VWR-Prolabo, 99%), methanol (VWR-Prolabo, 99%), POCl_3_ (Panreac, 99%), pyridine (Merck, 99%), sodium chloride (VWR-Prolabo, 99%), ((benzyloxy)carbonyl)arginine (TCI 97%), dichloromethane (VWR-Prolabo, 99%), tetrahydrofuran (VWR-Prolabo, 99%), *N*,*N*'-diciclohexilcarbodiimide (Alfa Aesar, 99%), tris(hydroxymethyl)aminomethane (Sigma-Aldrich, 99.8%), dimethyl sulfoxide (Scharlau, 99.9%), trypsin from bovine pancreas (Sigma-Aldrich, 90–100%), Z-L-Arg-7-amido-4-methylcoumarin hydrochloride (SUBS, Sigma-Aldrich, 98%), 7-amino-4-methylcoumarin (TCI, 98%). Azo-bis-isobutyronitrile (AIBN, Aldrich, 98%) was recrystallized twice from methanol. All the used solvents were of spectroscopic or equivalent grade and were used without further purification.

### Instrumentation

Enzymatic assays were conducted in buffer 0.1 M Tris–HCl pH 7.5 at 30 °C. Fluorescence was measured with 360/40–460/40 nm excitation/emission filters, using a Synergy HT microplate reader (BioTek®). Quantum yields were measured in a FLS980 Edinburgh Spectrometer equipped with an integrating sphere. The measurement conditions for all samples were: slits widths Δλ_exc_ = 2 nm, Δλ_em_ = 0.15 nm step = 0.2 nm, dwell = 0.3 s in triplicate. All data were measured at 25 °C ± 0.1 °C.

Infrared spectra (FTIR) were recorded with an FT/IR-4200 FT-IR Jasco Spectrometer with an ATR-PRO410-S single reflection accessory. High-resolution electron-impact mass spectrometry (EI-HRMS) was carried out on a Micromass AutoSpec Waters mass spectrometer (ionization energy: 70 eV; mass resolving power: > 10 000). ^1^H and ^13^C{^1^H} NMR spectra were recorded with a Bruker Avance III HD spectrometer operating at 300 MHz for ^1^H, and 75 MHz for ^13^C, using deuterated solvents like dimethyl sulfoxide (DMSO-*d*_*6*_) or deuterated chloroform (CDCl_3_) at 25 °C.

Thermal and mechanical properties of the material were measured using thermogravimetric analysis (TGA, 10–15 mg of the sample under synthetic air and nitrogen atmosphere with a TA Instruments Q50 TGA analyzer at 10 °C/min), differential scanning calorimetry (DSC, 10–15 mg of the sample under a nitrogen atmosphere with a TA Instruments Q200 DSC analyzer at 20 °C/min).

The powder X-ray diffraction (PXRD) patterns were obtained using a Bruker D8 Discover (Davinci design) diffractometer operating at 40 kV, using Cu(Kα) as the radiation source, and a scan step time of 2 s. Each spectrum was acquired from 5° to 70°, using a step size of 0.05° (2θ). Wavelength of X-ray radiation was 1.54060 nm, with an intensity of 30 mA.

Absorption and fluorescence spectra were obtained with a Cary 5000 spectrophotometer and a Horiba-Jobin-Ivon SPEX Fluorolog 3.22 spectrofluorometer, respectively, in a 10 mm quartz cuvette. All fluorescence spectra were corrected for the wavelength response of the system. The absorption of the solutions was kept under 0.1 at the excitation wavelength to avoid reabsorption and inner filter effects^47,48^.

Fluorescence decays were obtained using a nanosecond time-correlated single photon counting (ns-TCSPC) apparatus previously described^49^. The excitation source consists of a IBH 339 nm nano-led. Temperature control was achieved using a home-built system based on cooled nitrogen (for low temperature) and electric heating. Alternate acquisition of the sample and scattering solutions (aqueous Ludox solution) were obtained until 2–5 kCounts were reached. Fluorescence decay curves were deconvoluted using the experimental instrument response function signal collected with the scattering solution. The deconvolution procedure was performed using the modulation function method, as implemented by G. Striker in the SAND program, and previously reported in the literature^50^.

Samples for gel permeation chromatography (GPC) were weighed using an analytical scale. A measured amount of the eluent was added to set the concentration to 2–3 g/L. The samples dissolved at room temperature and were filtered before injection (filter pore size: 1 μm). Dimethylacetamide containing 0.05% of LiBr was used as eluent. Columns: PSS GRAM, 10 μm, combination high, 8 mm × 950 mm. Flow Rate: 1.0 ml/min. Injection System: Agilent-SECcurity Autosampler. Temperature: 70 °C. Detector: PSS-SECcurity DRI. PSS Eta2000 Differential viscosity detector. The size-exclusion chromatography column was calibrated with polystyrene calibration standards within the separation range of the column.

Isothermal titration calorimetry (ITC) measurements were performed using a microcalorimeter (VP-ITC MicroCal Inc., Malvern, UK) equipped with two cells, one cell for sample (SUBS, MONO-SUBS, and POLY-SUBS at concentration 0.1 mM) and another one for reference, with volumes of 1.436 mL. The titrant syringe was filled with 280 µL of a trypsin solution (0.1 μM) that was step-by-step added into the sample cell (10 μL aliquots were added every 30 min). All the solutions were degassed for 10 min at 25.0 ± 0.1 °C in a vacuum pump prior to the experiments to avoid blistering in the syringe or in the calorimetric cells during the experiment. Calorimetric measurements were performed at 25.000 ± 0.001 °C, with a constant stirring at 199 rpm in the sample cell.

### Synthesis of the polymerizable substrate benzyl (5-guanidino-1-oxo-1-((2-oxo-4-(2-oxo-2-((4-vinylphenyl)amino)ethyl)-2H-chromen-7-yl)amino)pentan-2-yl)carbamate (MONO-SUBS)

**Figure 2:**
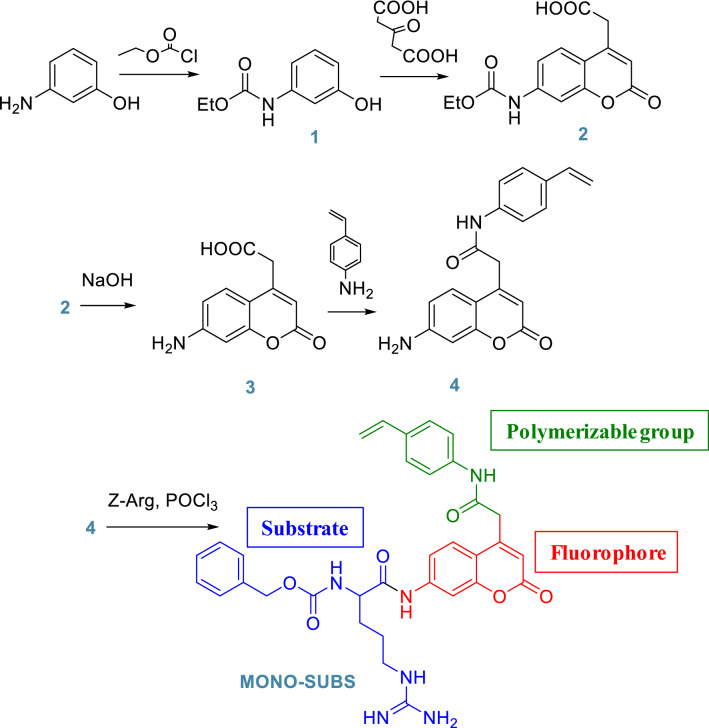
Synthetic route for the preparation of MONO-SUBS.

In Fig. 2, we depict the followed synthetic route, of which intermediates 1, 2, and 3 were synthesized and characterized as depicted in the bibliography^51,52^.

*Synthesis of 2-(7-amino-2-oxo-2H-chromen-4-yl)-N-(4-vinylphenyl)acetamide monomer (4). *A mixture of 2-(7-amino-2-oxo-2H-chromen-4-yl)acetic acid (3) (3.94 g, 17,98 mmol), *N*,*N*'-diciclohexilcarbodiimide (3.98 g, 18.85 mmol), 4-aminostyrene (2.1 mL, 2.14 g, 17.98 mmol) and 200 mL of THF was stirred overnight in a pressured flask at 45 °C. After that, the mixture was filtered, and the solvent was removed under reduced pressure. Finally, the solid was washed with 100 mL of DCM. Yield: 50%.^1^H NMR (300 MHz, DMSO-*d*_6_) δ (ppm) = 10.34 (s, 1H), 7.58 (d, J = 8.6 Hz, 2H), 7.48 (d, J = 15.4, 1H), 7.43 (d, J = 8.6 Hz, 2H)6.67 (dd, J = 17.7, 11.0 Hz, 1H), 6.57 (d, J = 8.7 Hz, 1H), 6.45 (s, 1H), 6.16 (s, 2H), 6.02 (s, 1H), 5.74 (d, J = 17.6 Hz, 1H), 5.18 (d, J = 11.7 Hz, 1H), 3.82 (s, 2H).^13^C NMR (75 MHz, DMSO-*d*_*6*_,) δ (ppm) = 167.24, 161.11, 156.14, 153.59, 151.52, 139.02, 136.54, 132.90, 127.08, 126.78, 119.64, 113.39, 111.70, 109.33, 108.75, 99.06. HRMS (EI) *m/z* [M + H]^+^ calc.: 321.1234; found: 321.1236. Further information in Figure S1, SI-Section S1.

*Synthesis of benzyl (5-guanidino-1-oxo-1-((2-oxo-4-(2-oxo-2-((4-vinylphenyl)amino)ethyl)-2H-chromen-7-yl)amino)pentan-2-yl)carbamate (MONO-SUBS).* A round bottom flask was placed in an ice-salt bath at − 15 °C (120 g of ice, and 40 g of NaCl), and a mixture of 4 (808 mg, 2.52 mmol), ((benzyloxy)carbonyl)arginine (Z-L-Arg, 777 mg, 2.52 mmol) and pyridine (12 mL) was prepared. Finally, POCl_3_ (415 mg, 2.7 mmol) was added dropwise to the mixture, and the resulting solution was stirred for 1 h at − 15 °C, and overnight at room temperature. Water was added to the flask until a white solid appeared, and the solution was acidified with 4% HCl. The solid was filtered and washed several times with water and once with methanol. Next, the crude product was treated with boiling ethanol (150 mL), filtered, and the solvent was removed under reduced pressure for obtaining the pure product as a yellowish solid. Yield: 40%. ^1^H NMR (300 MHz, DMSO-*d*_6_) δ (ppm) = 10.70 (s, 1H), 10.53 (m, 1H), 7.87–7.29 (m, 14H), 6.63 (m, 1H), 6.40 (M, 1H), 5.71 (d, 1H), 5.20 (m, 2H), 4.36 (m, 4H), 4.20 (s, 1H), 4.00 (m, 2H), 3.11 (m, 2H), 1.62 (m, 4H). HRMS (EI) *m/z* [M + H]^+^ calc.: 611.2613; found: 611.2616. Further information in Figure S2, SI-Section S1.

### General procedure for the synthesis of the polymers POLY-SUBS and POLY-(4)

Polymers were prepared by radical co-polymerization of the hydrophilic monomer VP with the synthesized monomers (MONO-SUBS and **(4)**) in a 99.5/0.5 molar ratio, respectively, to give POLY-SUBS and POLY-**(4)**, respectively. This molar ratio turned out to be the best for measuring enzyme activity, as we described in Figure S3, SI-Section S2. First, 0.09 mmol of the synthesized monomer (MONO-SUBS or **(4)**) and 17.94 mmol of VP were dissolved in DMF (9 mL), and the solution was added to a round-bottom pressure flask. Subsequently, radical thermal initiator AIBN (148 mg, 0.9 mmol) was added, and the solution sonicated for 10 min. Then, it was heated at 60 °C overnight, under a nitrogen atmosphere, without stirring. The solution was then dropwise added to diethyl ether (100 mL) with magnetic stirring, yielding the desired product as a white precipitate. Finally, polymers were purified in a Soxhlet apparatus with diethyl ether as the washing solvent for eliminating DMF traces. Yield ≈ 35%. Further information in SI-Section S3.

Two additional polymers were synthesized following the same procedure, specially oriented to the study of the protective environment exerted by polymeric chains. The first, a homopolymer, using only VP as the monomer (PVP). A homogeneous physical mixture of compound **(4)** and the homopolymer PVP was carried out in a vortex, mimicking POLY-**(4)** (weight of **(4)** to PVP like feed ratio of **(4)** to VP in the synthesis of POLY-**(4)**). The second, a co-polymer with a molar ratio 98/2 (VP/**(4)**).

### Measurement of enzymatic activity and kinetic parameters of different systems

**Figure 3:**
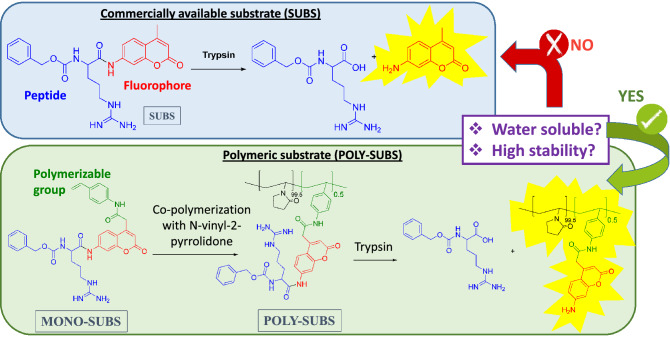
Chemical structures of SUBS, MONO-SUBS and POLY-SUBS.

To minimize errors, three systems shown in Fig. 3 (SUBS, MONO-SUBS and POLY-SUBS) were studied simultaneously and under the same conditions. The enzymatic activity and kinetic parameters were measured every week for 35 weeks to determine the stability of the different substrates, storing them both at room temperature and refrigerating at 4 °C. Enzymatic activity of commercial trypsin (EC 3.4.21.4) was tested in vitro using SUBS, MONO-SUBS and POLY-SUBS as substrates. Assays were carried out in 96-well microplates. Basically, 100 nM trypsin solutions were incubated with each substrate at a final concentration of 25 μM using the buffer mentioned above with 10% of DMSO, and the fluorescence enhancement was recorded over 2 h.

To determinate the kinetic parameters of the reaction, 200 nM of trypsin was incubated with different substrate concentrations ranging from 15 to 200 μM (15, 25, 50, 100, 125, 150, 200 μM) for each type of studied substrate (SUBS, MONO-SUBS and POLY-SUBS) and the fluorescence was recorded every 30 s for 10 min. Initial velocities were determined from the linear part of the curve and converted to the amount of hydrolyzed substrate per unit of time (μM/min).

The systems were calibrated with known amounts of AMC, compound **(4)** or POLY-**(4)**. Specific enzymatic activity was represented as nmol of substrate hydrolyzed/min/mg of protease.

The experiments were repeated after 1, 2, 3, 4, 5 and 35 weeks, storing all substrates both at room temperature and 4 °C. All assays were carried out by triplicate and blanks were used to account for spontaneous breakdown of substrates.

Usually, in this type of study in which a new substrate is characterized, the specific activity (expressed in nmol/min/mg of enzyme) and different kinetic parameters are calculated. Kinetic parameters of trypsin (Table 1) were calculated using the Michaelis–Menten equation in OriginPro Program.

**Table 1:** Kinetic parameters on day 0 of trypsin using different fluorogenic substrates.

Parameter	Provided information	SUBS	MONO-SUBS	POLY-SUBS
Specific activity (nmol/min/mg of enzyme)	Determines how efficiently an enzyme converts substrates into products. The higher value, the higher preference of the enzyme for the substrate	20.4 ± 0.6	34 ± 3	74 ± 2
v_max_ (µM/min)	Reaction rate at which the enzyme catalyzes the reaction that occurs when all active sites of the enzyme are saturated with substrate	1.18 ± 0.08	27 ± 6	0.44 ± 0.01
K_M_ (µM)	Substrate´s concentration which allows the enzyme to achieve ½ Vmax. The higher value, the lower affinity of the enzyme for the substrate	0.41 ± 0.05	15 ± 4	1.30 ± 0.07
K_cat_/K_M_ (µM^−1^/min^−1^)	Specificity of the enzyme for the substrate. The higher value, the higher the frequency of encounter between the enzyme and the substrate	14.5 ± 0.4	9.4 ± 0.3	1.68 ± 0.04

Data are means ± SE of three replicates.

## Results and discussion

### Polymer characterization

#### X-ray powder diffraction (XRD) analysis

The X-ray diffraction spectra of POLY-(**4**) and POLY-SUBS show two diffused halo peaks in the amorphous region, 11.38° and 21.58° 2θ values for POLY-(**4**) and 12.01° and 20.42° for POLY-SUBS (SI-Section S3). These peaks correspond to that described in the literature for pure PVP^53^, with slight variations due to the pendant motifs derived from the use of monomers (**4**) and MONO-SUBS in the preparation of the copolymers. The first diffuse peak (2θ ~ 11.5°) is due to interactions of C–C polymer main chains, while the second peak (2θ ~ 21°) is related to inter- and intramolecular interactions between the pyrrolidone rings^54^.

#### Gel permeation chromatography

The molecular mass study of POLY-(**4**) and POLY-SUBS was carried out by GPC, giving similar results for both polymers, in agreement with the use of same conditions and 99.5 mol% of VP in both in the polymer preparation. The molecular weight distribution curves (Mw) showed bands centered at 10.2–10.3 KDa (see SI-Section S3). These polymers were specially designed to have relatively low molecular mass, as finally obtained, for having very good solubility. Additionally, absolute molecular weights were calculated for both polymers in two ways: differential viscosity assays and universal calibration.

### Characterization of substrates

#### Characterization of substrates´ kinetic parameters

The enzymatic activity and kinetic parameters at the beginning of the study are depicted in Table 1. The commercial substrate SUBS has the lowest K_M_ value, and therefore the highest affinity of the enzyme for that substrate. This was predictable since this substrate has been used for many years, and many authors have reported good results^40,55,56^. However, it was not foreseeable that a small modification of the molecule, such as MONO-SUBS, would cause such a great decline in K_M_. In fact, K_M_ is 36 times higher than that of SUBS, so the enzyme's affinity for MONO-SUBS is much lower. Surprisingly, this negative effect disappears almost completely upon co-polymerizing MONO-SUBS with VP, i.e., when using the POLY-SUBS substrate. Thus, POLY-SUBS has a K_M_ value of the same order as SUBS. This may come from the protective environment generated by polymer chains (mainly comprised of structural units coming from the co-polymerization of VP) on the enzymatically degradable motifs, as shown in the graphical abstract.

K_cat_/K_M_ value in POLY-SUBS is notably lower compared to the rest of assayed substrates. Our interpretation is that the frequency of encounter of the enzyme and the substrate is lower due to the protective environment generated by polymer chains, making the enzymatically degradable motifs less accessible for the enzyme. However, once the enzyme overcomes the protective environment of POLY-SUBS, this substrate presents the best data for the trypsin specific activity, showing that the reaction between trypsin and motifs occurs in a highly favored way. This fact encouraged us to study the reaction´s equilibrium constant by isothermal titration calorimetry using the three substrates.

#### Characterization of the interaction between trypsin and substrates by isothermal titration calorimetry.

**Figure 4:**
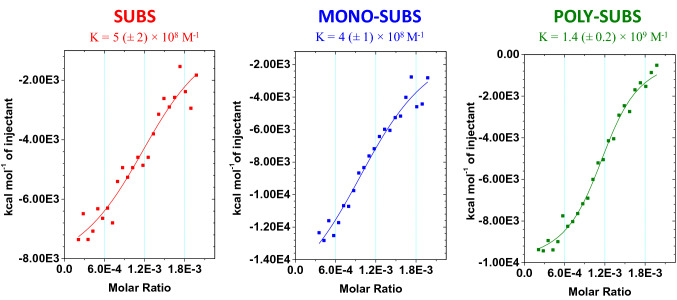
Study of the reaction between trypsin and three different substrates by isothermal titration calorimetry. The assays were carried out at 25.00 °C, using substrates and trypsin concentrations of 0.1 mM and 0.1 μM, respectively, in DMSO:Buffer pH7 (10:90). Graphs show the equilibrium constant with calculated errors.

The hypothesis raised in the previous section was verified by isothermal titration calorimetry, a widely used method to analyze binding between trypsin and different ligands^57,58^. The addition of trypsin to substrates SUBS, MONO-SUBS and POLY-SUBS (from left to right in Fig. 4) lead to exothermic interactions, which decrease by increasing the concentration of trypsin. In order to have an assessment on the stability of interaction between the enzyme (E) and the substrates (S), assuming a 1:1 interaction, the binding constant, *K*, of the complex (E-S) can be written as

1 $$K = \frac{{\left[ {E - S} \right]}}{[E][S]}$$

where [*E*] and [*S*] represent the concentration of free (non-reacted) enzyme and substrate, respectively. From mass balance equations, the concentration of the complex E-S can be calculated from:

2 $$[E - S]^{2} - \left( {[E]_{T} + [S]_{T} + \frac{1}{K}} \right)[E - S] + [E]_{T} [S]_{T} = 0$$

where [*S*]_*T*_ and [*E*]_*T*_ represent the initial concentrations of trypsin and substrate, respectively^59^.

The heat produced in an experiment in which *n*_E-S_ moles of complex are formed is given by:

3 $$Q = n_{E - S} \cdot \Delta H$$

where Δ*H* is the molar enthalpy of binding and *n*_E-A_ is the amount of complex. In an ITC experiment, the heat generated by the *i*-th injection is given by the following Eq. ^60^:

4 $$Q_{i} = (n_{{E - S,i}} - n_{{E - S,i - 1}} ) \cdot \Delta H_{i} = (V_{i} [E - S]_{i} - V_{{i - 1}} [E - S]_{{i - 1}} \cdot \Delta H_{i}$$

where [*E-S*]_*i*_ is the concentration of the complex, as computed from Eq. (2) and *V*_*i*_ is the total volume of solution after injection *i*, respectively.

By fitting Eqs. (1 and 4) to the experimental data (solid lines in Fig. 4) the equilibrium constants are computed for all three systems, and the following K values are obtained: 5 × 10^8^, 4 × 10^8^ and 1.4 × 10^9^ M^−1^, for SUBS, MONO-SUBS and POLY-SUBS, respectively. The equilibrium constant for interaction between POLY-SUBS and trypsin is one order of magnitude higher than those found for other substrates. This evidence clearly confirms that polymeric chains allow the formation of a highly stable product, and a stronger interaction with the enzyme compared with SUBS and MONO-SUBS substrates.

#### Proof of concept. Long-term stability study.

**Figure 5:**
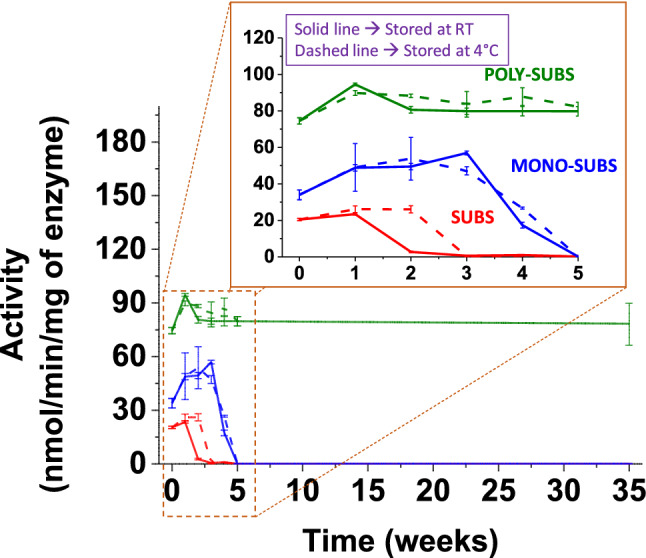
Stability study of three different substrates, i.e., SUBS, MONO-SUBS, and POLY-SUBS, stored at 4 °C and room temperature for 35 weeks.

After the preliminary study of the kinetic parameters, we set out to demonstrate the generated protective environment with a proof of concept, in which we repeated the same analysis after 1, 2, 3, 4, 5, and 35 weeks, with the substrates stored at room temperature and at 4 °C. In this way, the protective environment generated in POLY-SUBS should make the substrates more stable and less sensitive to aging, light and temperature changes. Thus, we have monitored the stability of different substrates during 35 weeks by following the trypsin´s activity with SUBS, MONO-SUBS and POLY-SUBS, since there is no activity if the substrate is damaged. However, as shown in Fig. 5, this proof of concept confirms the low stability of the commercial substrate, SUBS, both stored at room temperature and at 4 °C. In fact, as of the second week, the activity data could not even be calculated.

Trypsin activity was detectable using MONO-SUBS during the first four weeks. However, in the 4th week, the substrate suffered a very pronounced deterioration, which was translated into a total loss of activity from the 5th week on.

On the other hand, trypsin activity was detectable using POLY-SUBS after 35 weeks without observable substrate damage, regardless of the storage temperature. These values represent a lifetime improvement of at least 600% and 700% related to MONO-SUBS and SUBS, respectively.

### Fundamentals of the protective environment exerted by polymer chains

The basis of the protective environment generated by polymer chains over the contained enzymatically degradable motifs was the most important challenge of this work. Compound **(4)** and POLY-**(4)** were chosen for this study since they offer great characterization possibilities, including fluorimetry. Therefore, the study described below was carried out by thermogravimetry fluorimetry and UV–Vis spectroscopy. In this way, once the fundamentals of the generated protective environment have been established, these can be extrapolated to POLY-SUBS, and other future polymers containing enzymatically degradable motifs. The subsections below have been described chronologically to facilitate understanding the followed process. This process began with a spectroscopic study (fluorescence and UV–Vis) that indicated the possible existence of the protective effect, and ended with the definitive proof reached with the thermogravimetric study.

#### Photophysical measurements

Neither the modification of SUBS´ chemical structure with a polymerizable group nor the co-polymerization of this new compound with VP significantly affects the resulting fluorescence quantum yield, i.e., the fluorescence efficiency of the sample solutions. The fluorescence quantum yields for compound **(4)** and POLY**-(4)** are found very high, reaching values of 86.58% and 87.62%, respectively.

However, we found great differences when studying another key parameter associated with photophysical characterization. Indeed, time-resolved fluorescence (TRF) measurements provide an additional tool to investigate the excited state behavior of the systems deeply. Moreover, time-resolved measurements is a handy tool that some authors have used to demonstrate similar protection environments to those we have observed in our study^61^. For example, Okabe et al. showed that the fluorescence lifetime increases when a fluorophore is protected with a thermosensitive polymer^62^. Additionally, Królicki et al. carried out similar experiments, in this case studying the protection of a coumarin based fluorophore when solvated with different solvents, and they observed the corresponding increase in the fluorescence lifetime decays τ_F_^63^.

**Figure 6:**
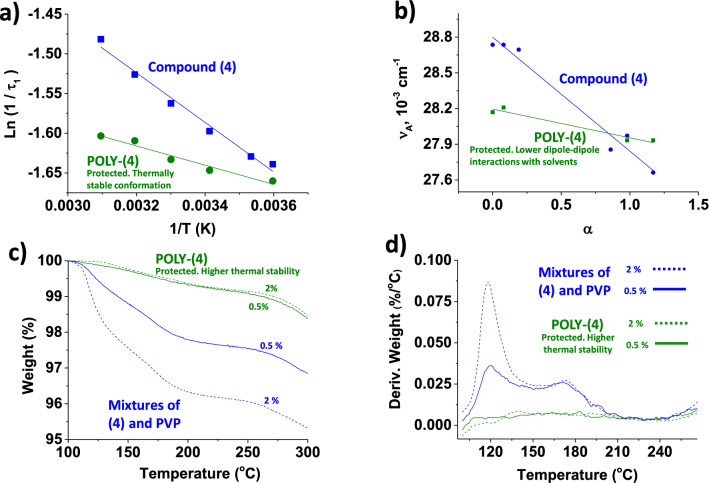
**(a)** Logarithm representation of an Arrehnius type plot for the reciprocal of τ1 with temperature for solutions of compound **(4)** and POLY-**(4)** in DMSO:H_2_O (10:90). **(b)** Solutions of POLY-**(4)** and compound **(4)** were prepared in different solvents (water, methanol, ethanol, acetonitrile, and acetone) and measured by UV–Vis spectroscopy. The wavenumber corresponding to the maximum absorbance of each spectrum is represented against the acidity component of each solvent (α). The greater slope, the more intensive dipole–dipole interactions of the chromogenic units with solvents **(c)** Thermograms and **(d)** derivative weight loss for POLY-**(4)** (green curves) and for mixtures of compound **(4)** with poly(*N*-vinylpyrrolidone) (blue curves), at molar ratios of the fluorescent units of 0.5 and 2 (solid and dashed, respectively). Heating rate 10 °C/min and nitrogen atmosphere.

The fluorescence decay times, and pre-exponential factors obtained for both systems in water (10% DMSO) are presented in SI-Section S4, Table S1. The time-resolved fluorescence decays are found to fit, for compound **(4)**, to a mono-exponential decay law (with τ_1_ values in Table S1), clearly indicating that the fluorescence decay of this species does not involve any additional process when isolated in solution. However, incorporation of the coumarin moiety into the polymer leads to a double exponential decay law with two components (τ_1_ and τ_2_ in Table S1). From a qualitative point of view, this can be justified by the fact that in POLY-(**4**) the coumarin chromophore probes two different environments in contrast with (**4**) in solution. Moreover, the fact that the major contribution (as given by the fractional contributions %Ci) is still associated to the longer-lived component (τ_2_), with more than 90% of the contribution, shows that the decay is mainly due to the decay of the isolated coumarin. The additional shorter decay component results from the random nature of the labelling which introduces regions more densely rich in coumarin with others less rich^64^. Thus, our current interpretation is that the random polymerization of POLY-**(4)** leads to two local environments for the coumarin probe: (a) one where the coumarin are submitted to a quenching which result from a more densely labeled region of the polymer, mirrored by the short-lived species (τ_1_) contribution, where there is any energy transfer mechanism involving different coumarins at short interaction distances; and (b) a long-lived one (τ_2_) associated with more separated and isolated coumarins. Although these results indicate a greater abundance of isolated coumarins along the polymer, as opposed to areas with coumarins in close proximity to each other (due to a higher contribution of (2), this interpretation is non-conclusive itself regarding the protective play of the polymer. Thus, further studies were carried out, and fluorescence decays as a function of temperature (Table S1) were performed to unveil the role of the polymer. For compound (**4**), an increase in the temperature is followed by a decrease in the fluorescence decay time, τ_1_, due to the increase in the radiationless contribution. However, and in line with previous findings, almost no variation is observed for POLY-(**4**) (Fig. 6a and Table S1), thus showing that the polymer can somehow be led to a level of protection of the coumarin moieties that makes them insensitive to temperature changes, within the investigated temperature range. This.

#### Solvation study by UV–Vis spectroscopy

This study might be helpful to characterize dipole–dipole interactions between a colored/fluorescent molecule and the solvent^33,65^. Our hypothesis relies on the idea that if colored/fluorescent units of compound (**4**) are protected in POLY-(**4**), the interactions with the solvent will be lower. Therefore, the absorption spectrum of compound (**4**) and POLY-(**4**) in different solvents was measured (water, methanol, ethanol, acetonitrile, and acetone). Each of these solvents has a unique polarity, acidity, and basicity character, determined by a tabulated parameter (see Table S2, SI-Section S5). First, this study demonstrated that the most relevant parameter of this system is the acidity component of each solvent. Second, in agreement with the TRF measurements, when plotting absorbance spectrum´s shifts (in wavenumbers, cm^−1^) against the acidity parameter of each solvent, we observe that the influence of the solvent is much higher with (**4**) than with POLY-(**4**), since to a higher value for the slope, corresponds to an increment on the interactions of the colored/fluorescent motifs with the solvent. Additionally, since the shift in the wavelength of the absorbance spectra occurs from lower to higher values when solvents´ acidity increases, the interactions of the coumarin derivatives with the solvent are mainly established through the carbonyl group, as determined by X. Liu et al.^65^ Results are shown in Fig. 6b, and support the commented protective environment generated by polymer chains.

#### Thermogravimetry

After analyzing the UV–Vis and the spectroscopy results, we decided to carry out a thermogravimetric study. In this case, the hypothesis was that the protective environment generated by polymer chains would make the thermal degradation of the fluorescent units different from that of discrete molecules of compound (**4**). Therefore, the thermal degradation of fluorescent units covalently anchored to the main chain of the polymer (POLY-(**4**)) and not-anchored was studied.

Figure 6c and d show the results obtained, in which an extraordinary increase in thermal stability is observed in POLY-(**4**). Considering the fluorophore concentration in both tests (0.5 mol%), the tests were repeated at a higher fluorophore concentration of 2% to obtain additional information. In the case of the mixture of compound (**4**) and PVP (not anchored motifs), the weight loss observed below 300 °C is much greater, due to the greater amount of compound (**4**) in the mixture of solids, as expected. However, in POLY-(**4**) (anchored motifs, 2% mol), the thermal stability below 300 °C remains the same as in the previous case, where the anchored motifs were 4 times lower, confirming the observed protective environment again.

## Conclusions

Fluorescent peptide substrates have extraordinary potential in the study and analyses of enzyme activity, and especially for determining the activity of proteases, but have the drawback of their thermal and light instability, specifically in solution, and also the lack of solubility in water. In this work, we have overcome these drawbacks, deeply improving their environmental stability, extending the lifetime of these substrates at least by 700%, and the same time providing solubility in water, by simply preparing co-polymers having the fluorescent substrate chemically anchored to the polymer backbone, mainly using the commercial monomer *N*-vinyl-2-pyrrolidone and a small amount of a vinylic monomer containing the substrate. Thus, these sensory materials have the significant advantage in laboratory used of rendering stable results in fully aqueous solutions, regardless the thermal and light history. Regarding the commented advantages, the polymer with both pendants substrate and fluorescent motifs has been tested with trypsin, but we envisage a similar behavior with other peptides and enzymes.

Moreover, giving the increment of stability provided by the polymeric nature of the substrate, and its activity, we are working on stable sensory materials for the visual control of diseases, coatings to prevent the proliferation of microorganisms in surfaces and textiles, for the detection of viruses, e.g., in masks to detect viruses, etc.

## Supplementary Information

Supplementary Information.

## Data Availability

The datasets used and/or analysed during the current study available from the corresponding author on reasonable request.
