# Supplementary material for: The role of polymeric chains as a protective environment for improving the stability and efficiency of fluorogenic peptide substrates
Source: Sci Rep. 2022 May 25;12:8818. doi: 10.1038/s41598-022-12848-4 (PMC9132916; doi:10.1038/s41598-022-12848-4)
Supplement: Supplementary file 1 — Supplementary Information. [file 41598_2022_12848_MOESM1_ESM.pdf]

Electronic Supplementary Information (ESI)

THE ROLE OF POLYMERIC CHAINS AS A PROTECTIVE ENVIRONMENT FOR IMPROVING THE STABILITY AND EFFICIENCY OF FLUOROGENIC PEPTIDE SUBSTRATES

Ana ARNAIZ[a], Marta GUEMBE-GARCÍA[a], Estefanía DELGADO-PINAR[b], Artur VALENTE[b], Saturnino IBEAS[a], José M. GARCÍA[a], Saúl VALLEJOS[a*]

[a] Departamento de Química, Facultad de Ciencias, Universidad de Burgos, Plaza de Misael Bañuelos s/n, 09001 Burgos, Spain
[b] University of Coimbra, CQC, Department of Chemistry, Rua Larga, 3004-535 Coimbra, Portugal
* Corresponding author: Dr Vallejos (svallejos@ubu.es)

Table of contents

S1. Characterization of monomers 2
S2. Determination of the optimum molar ratio of the copolymer 4
S3. Characterization of polymers 5
S4. Time resolved fluorescence data 7
S5. π*, α, and β parameters for different solvents 7

S1. Characterization of monomers

- *Characterization of 2-(7-amino-2-oxo-2H-chromen-4-yl)-N-(4-vinylphenyl)acetamide* **(4)**

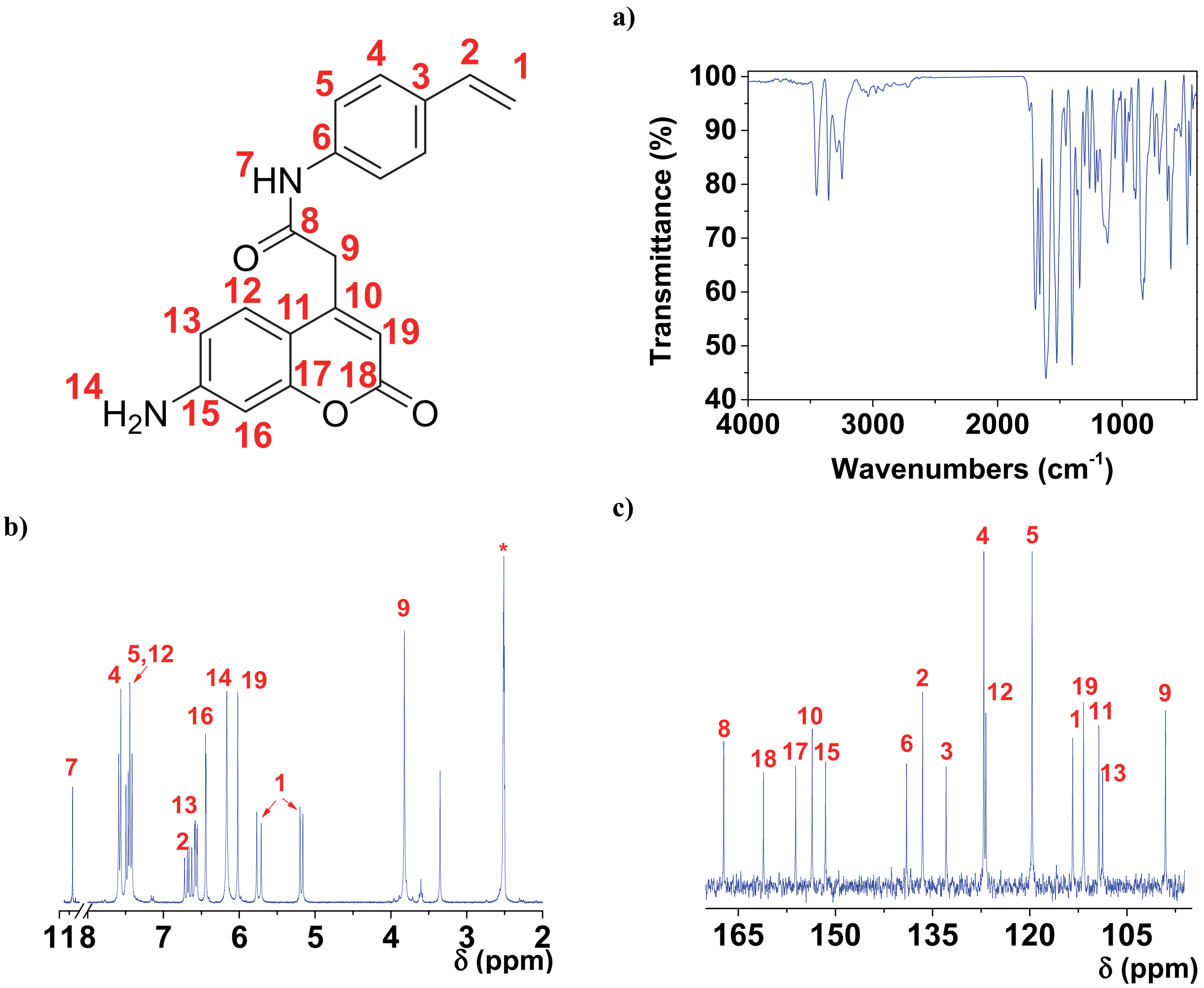

Figure S1. Characterization of **(4)** by (a) FTIR, (b) ^{1}H NMR, and (c) ^{13}C NMR spectroscopies (* = solvent signal, DMSO-d_6).

- *Characterization of benzyl (5-guanidino-1-oxo-1-((2-oxo-4-(2-oxo-2-((4-vinylphenyl)amino)ethyl)-2H-chromen-7-yl)amino)pentan-2-yl)carbamate* **(MONO-SUBS)**

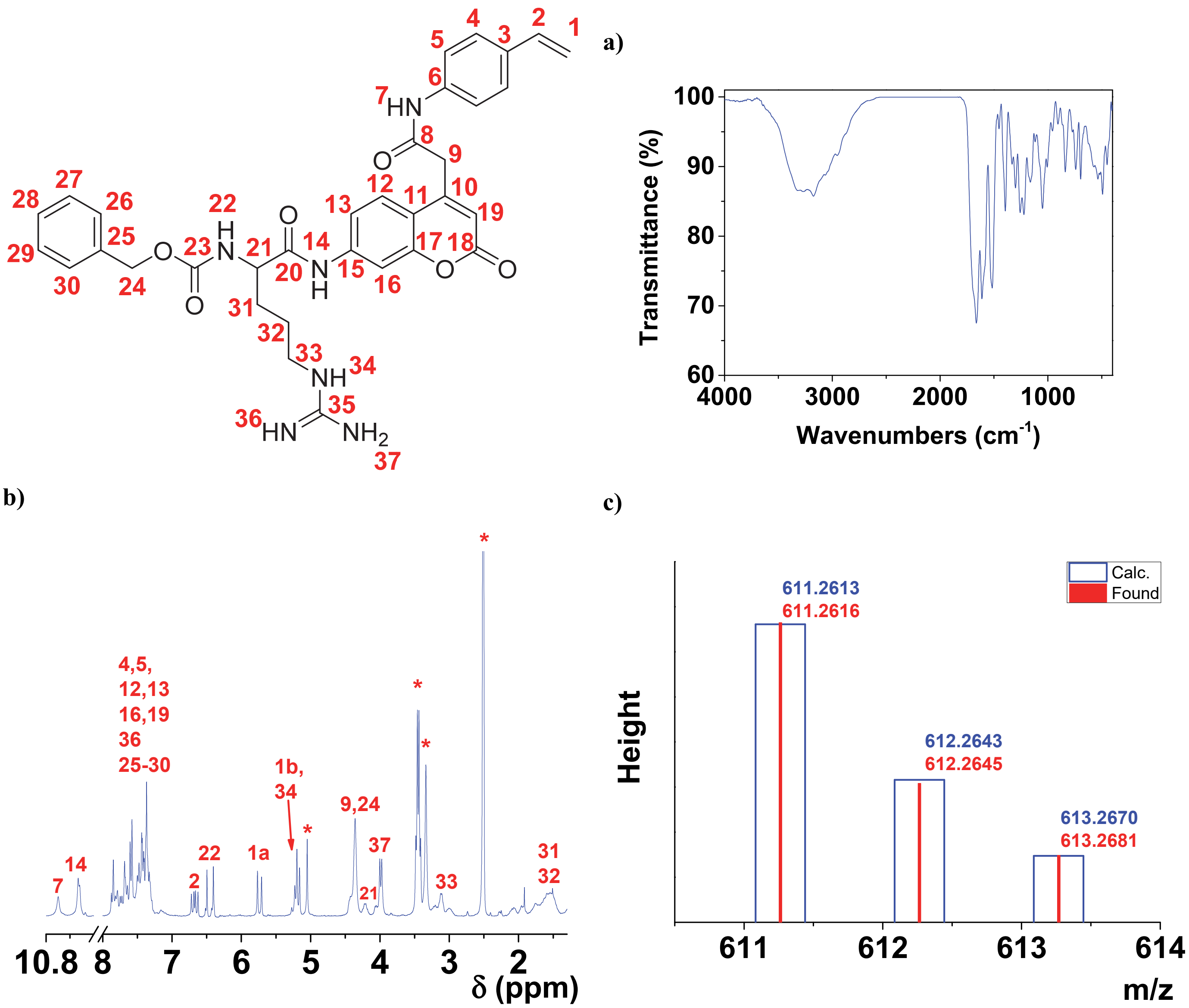

Figure S2. Characterization of **MONO-SUBS** by (a) FTIR, (b) ^{1}H NMR, and (c) EI-HRMS spectroscopies (* = solvent signal, DMSO-d_6).

S2. Determination of the optimum molar ratio of the copolymer.

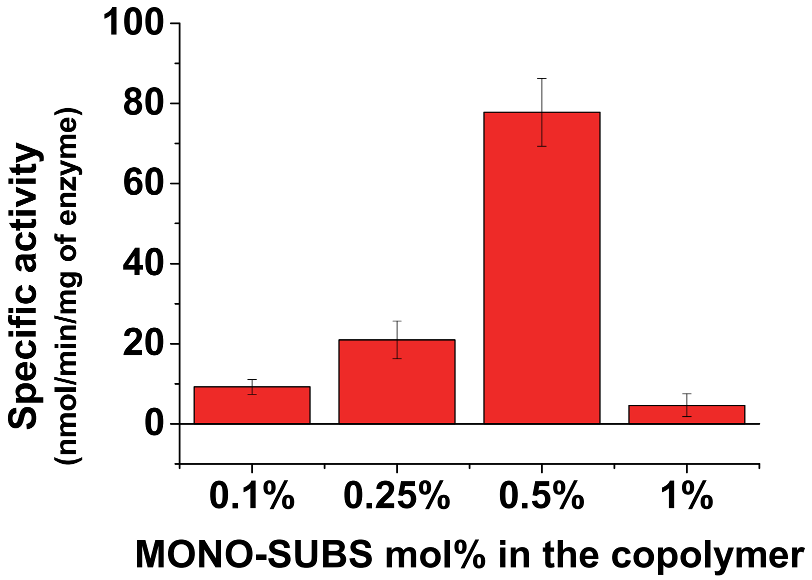

Figure S3. Specific trypsin activity (expressed as nmol of substrate hydrolyzed/min/mg of trypsin) of different copolymers with MONO-SUBS mol% ranging from 0.1 % to 1 %.

S3. Characterization of the polymers

a)

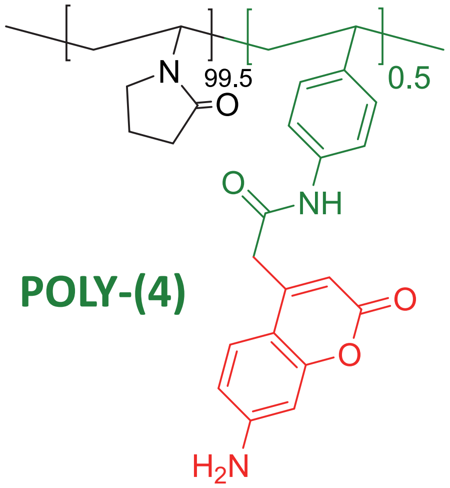

b)

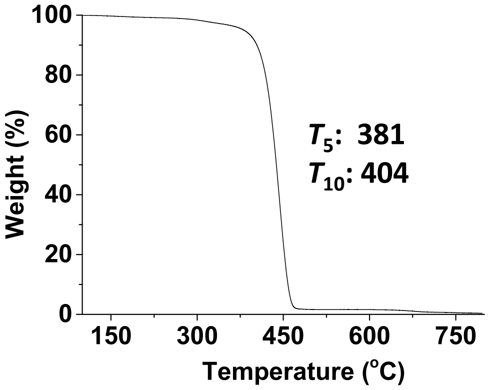

c)

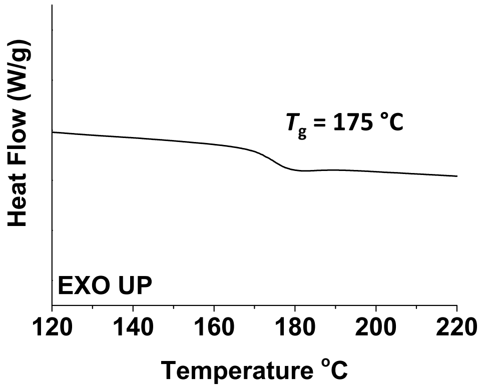

d)

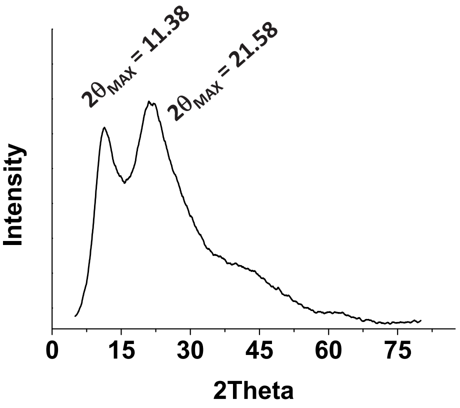

e)

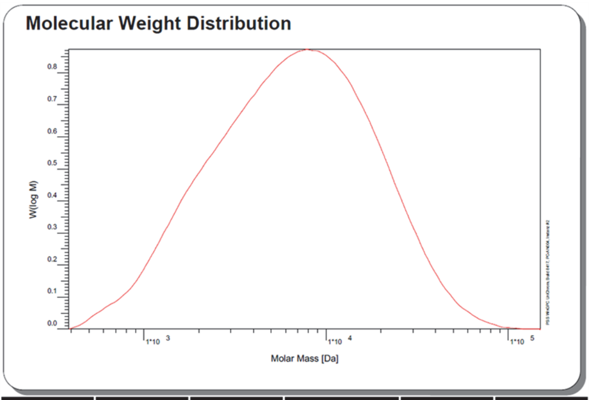

Mn (Da)	Mw (Da)	Mz (Da)	PDI (=Mw/Mn)	Vp (mL)	Mp (Da)
3990	10200	21100	2.56	29.22	7870

f)

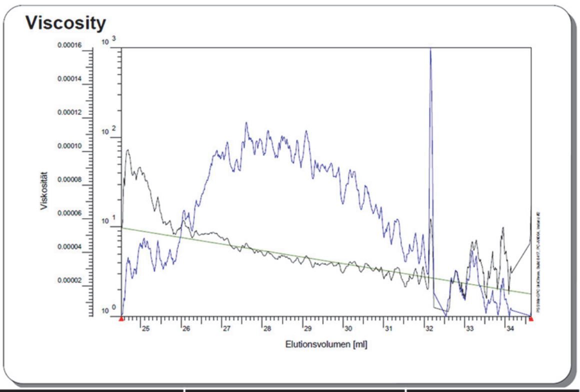

Mark-Houwink alpha	Mark-Houwink K	[η]
0.4549	0.1427 mL/g	4.65 cm^3/g

Figure S4. Polymer characterization: (**a**) chemical structure; (**b**) TGA curves at heating rate of 10 °C·min^{-1} under nitrogen atmosphere; (**c**) DSC curve at a heating rate of 20 °C·min^{-1} under nitrogen atmosphere; (**d**) PXRD spectra showing $2\theta_{MAX}$ values; (**e**) GPC chromatogram; (**f**) intrinsic viscosity vs volume.

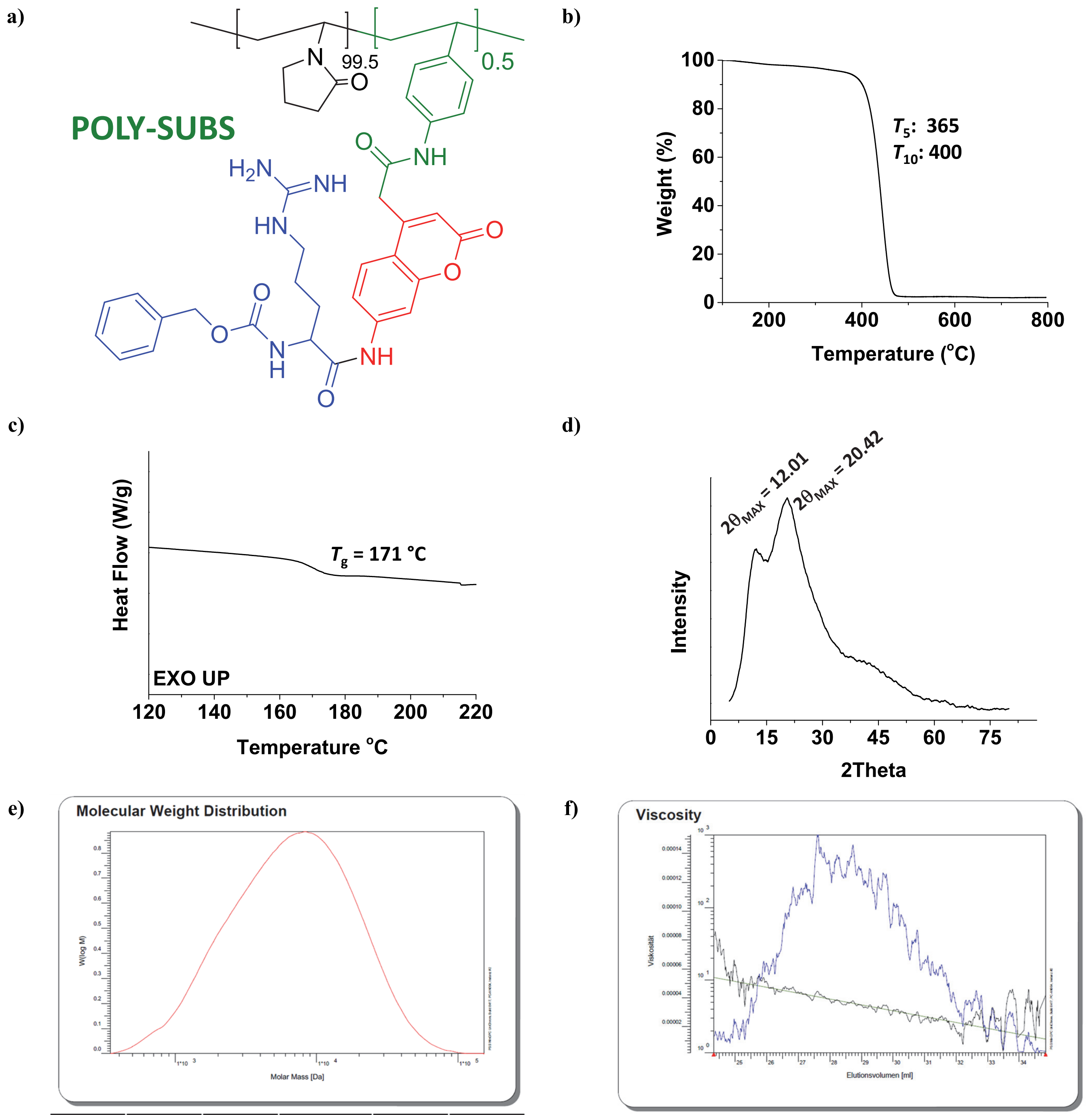

Mn (Da)	Mw (Da)	Mz (Da)	PDI (=Mw/ Mn)	Vp (mL)	Mp (Da)
4090	10200	20900	2.51	29.13	8300

Mark-Houwink Alpha	Mark-Houwink K	[η]
0.5699	0.0583 mL/g	4.47 cm^3/g

Figure S5. Polymer characterization: (**a**) chemical structure; (**b**) TGA curves at heating rate of 10 °C·min^{-1} under nitrogen atmosphere; (**c**) DSC curve at a heating rate of 20 °C·min^{-1} under nitrogen atmosphere; (**d**) PXRD spectra showing 2θ$_{MAX}$ values; (**e**) GPC chromatogram; (**f**) intrinsic viscosity vs volume.

S4. Time-resolved fluorescence data

The fluorescence intensity response with time, I(t), is given by Equation 1 with decay times τ_1 and τ_2, and pre-exponential factors (a_1 and a_2) mirroring the excited state concentrations at time zero:

$$I(t) = a_1 e^{-\frac{t}{\tau_1}} + a_2 e^{-\frac{t}{\tau_2}} \qquad (1)$$

An additional parameter obtained from the decays, the fractional contribution (C_i) of each species, allows a better interpretation of these results. The C_i, given in **Table S1**, for each species (1 and 2) is given by the following equation,[1]

$$C_i(\%) = \frac{a_i \tau_i}{\sum_{i=1}^{n} a_i \tau_i} \times 100 \qquad (2)$$

where n stands for the number of exponential terms, a_i the contribution of each exponential term at $t = 0$ and τ_i are the associated decay times.

Table S1. Time resolved fluorescence data (lifetimes, τ_i, pre-exponential factors, a_i, and chi-squared values, χ^2) obtained at different temperatures in DMSO:H_2O (10:90). λ_{exc} = 339 nm and λ_{em} = 454 nm.

	T (ºC)	τ1	τ2	χ^2	a1	a2	c1	c2
**Compound (4)**	5	5.15		1.47				
	10	5.1		1.24				
	20	4.94		1.13				
	30	4.77		1.1				
	40	4.6		1.19				
	50	4.4		1.27				
	**T (ºC)**	**τ1**	**τ2**	**χ^2**	**a1**	**a2**	**c1**	**c2**
**POLY-(4)**	5	1.27	5.26	1.24	0.238	0.762	0.07	0.93
	10	0.91	5.14	1.26	0.22	0.78	0.05	0.95
	20	1.53	5.19	1.33	0.236	0.764	0.08	0.92
	30	1.44	5.12	1.2	0.219	0.781	0.07	0.93
	40	1.33	5	1.06	0.239	0.761	0.08	0.92
	50	1.54	4.97	1.19	0.262	0.738	0.10	0.90

S5. π*, α, and β parameters for different solvents

Table S2. Obtained wavenumbers at the maximum of the UV-Vis curves of POLY-SUBS and compound **(4)** in different solvents. The table also shows tabulated π*, α, and β parameters for the Taft-Kamlet solvatochromic model, describing the polarity of the solvent, the acidity or ability to donate a proton to a hydrogen bond (HBD) and the basicity or ability to accept a proton from a hydrogen bond (HBA) respectively.

Solvent	Parameter			ν_A, 10^{-3} cm^{-1}	
	π*	α	β	POLY-(4)	Compound (4)
**Water**	1.09	1.17	0.47	27.93	27.66
**Metanol**	0.60	0.98	0.66	27.93	27.97
**Etanol**	0.54	0.86	0.75		27.86
**Acetonitrile**	0.75	0.19	0.40		28.69
**Acetone**	0.71	0.08	0.43	28.21	28.74
**Dioxane**	0.55	0.00	0.37	28.17	28.74

References

(1) Delgado-Pinar, E.; Costa, A. L.; Gonçalves, I. S.; Pineiro, M.; Pillinger, M.; Melo, J. S. S. de. Tuning the Behavior of a Hydrotalcite-Supported Sulfonated Bithiophene from Aggregation-Caused Quenching to Efficient Monomer Luminescence. *J. Phys. Chem. C* **2021**, *125* (15), 8294–8303. https://doi.org/10.1021/ACS.JPCC.1C00240.
